# Targeted questionnaires improve detection of early gastrointestinal symptoms in young children with Fabry disease

**DOI:** 10.1186/s13023-025-04168-3

**Published:** 2026-01-20

**Authors:** Anika Quillin, Hannah Waddel, Gwen Gunn, Jared Druss, Nadia Ali, William Wilcox, Dawn Laney

**Affiliations:** 1 Department of Human Genetics, Emory University School of Medicine, Atlanta, GA USA; 2 Department of Biostatistics and Bioinformatics, Emory University Rollins School of Public Health, Atlanta, GA USA

**Keywords:** Fabry disease, Gastrointestinal, Symptoms, Questionnaire, Pediatric, Onset, Rome III

## Abstract

**Background:**

Fabry disease (FD) is a multisystemic, progressive, X-linked genetic disorder caused by dysfunction of the enzyme α-galactosidase A. Symptoms commonly present in childhood in classic patients. Prior studies in classic patients suggest primary presenting features include gastrointestinal (GI) symptoms such as abdominal pain, bloating, and diarrhea. In this longitudinal study, we collected annual questionnaires and medical records from families of 29 children between 3 months and 9.5 years old, including the Rome III questionnaire. We compared symptom detection of abdominal pain, constipation, diarrhea, and bloating via three methods: the Rome III, a simple review of symptoms questionnaire, and pediatrician notes including a review of systems.

**Results:**

Both questionnaires elicited all GI symptoms more frequently than pediatrician notes (log rank test, *p* < 0.001). The two questionnaires had weaker agreement for constipation than other symptoms (per kappa statistic) and more similar detection at a younger age. After 24 months, the Rome III outperformed the simple review of symptoms questionnaire (Fisher’s exact test, *p* < 0.001). Pediatrician notes never recorded bloating or severe episodes of abdominal pain.

**Conclusions:**

Targeted questionnaires elicit early gastrointestinal symptoms in pediatric Fabry disease patients that would otherwise go unnoticed at a standard doctor’s appointment. Based on manual analysis of questionnaire data, a list of questions is suggested to support pediatricians of young patients with FD in recognizing GI symptoms. Use of more targeted and specific questions regarding GI symptoms is warranted in pediatric appointments of patients with FD, with age-appropriate expansion of the questions at 24 months of age. Early detection of symptoms in this population is critical as individual treatment plans are based on symptom onset and as newborn screening is expanding.

**Supplementary Information:**

The online version contains supplementary material available at 10.1186/s13023-025-04168-3.

## Background

Fabry disease (FD; OMIM 301,500) is a lysosomal storage disease caused by pathogenic variants in the *GLA* gene which result in a deficiency of the enzyme alpha-galactosidase A (α-gal A; EC 3.2.1.22), causing a build-up of glycosphingolipids such as globotriaosylceramide (GL3) [1]. There are classic and nonclassic phenotypes of FD with some genotype-phenotype correlation. Nonclassic FD is characterized by later symptom onset and higher baseline levels of α-gal A. Both males and females experience symptoms of this X-linked condition, although disease progression and impact are more uniform in males [2].

Symptoms of classic FD present during childhood. Previously reported median age of onset for males is six years [3, 4]. Symptoms in children under five years are less well-described but do occur [5]. Neuropathic pain and gastrointestinal (GI) symptoms are common in childhood [3, 5]. Recent research reports an average symptom onset of 18.5 months with common first symptoms of heat and cold intolerance, hypohidrosis, and GI symptoms [6]. Later in life, symptoms can progress to renal failure, cardiac and cerebrovascular disease.

In children with FD, the ability to detect symptoms early is essential because guidelines for starting enzyme replacement therapy (ERT) are based on symptom onset [7, 8]. Starting ERT before 18 years of age may prevent build-up of GL3 and associated inflammatory response, improving outcomes [3, 9]. Experts in the field have debated whether even earlier treatment would be beneficial [4, 10, 11]. Better symptom detection in children would inform both guidelines and individual treatment plans. Additionally, there is a need for systematic guidelines for newborn screening follow-up, as eight states currently perform newborn screening for FD [12]

.The earliest symptoms of FD described in the literature are GI-focused: abdominal pain, bloating, diarrhea, and alternating constipation and diarrhea [7]. Though they can greatly impact patients’ lives and present early in life, the non-specificity of GI symptoms poses a challenge. This is a current topic of discussion in the field, with multiple papers published within the last year questioning whether ERT should be initiated at an earlier age in response to GI symptoms and arguing that general practitioners should be giving more attention to the GI symptoms associated with FD [13–15]. Even though GI symptoms have been documented under the age of 4, current tools for assessing these symptoms focus on children age 4 and up [16].

As more consideration is given to this aspect of FD, it is critical that we look to the right tools to monitor GI symptoms in very young children with FD. Although in some countries, all patients with FD are monitored closely by a metabolic center at all ages, in other countries much of this monitoring will come down to generalist providers, because they see patients most frequently and not all FD patients choose to follow with or have easy access to a metabolic or genetics specialist. Physical exams and pediatrician notes routinely include evaluation of GI symptoms but may not provide the level of detail needed for this purpose. Questionnaires are another way to elicit information and document the information for metabolic specialists and families. One review of systems (ROS) questionnaire was developed by centers of excellence in Fabry disease and is now used clinically and routinely by these centers. Another option is to incorporate the Rome III criteria, which describe various functional gastrointestinal disorders. The extremely detailed pediatric Rome III questionnaire is validated for use in children starting at age four [17]. It can be used by family physicians and has been used previously in FD [6, 18]. Prior use in FD detected bloating as early as 11 months, chronic diarrhea as early as 23 months and chronic abdominal pain as early as 25 months [6], suggesting that the Rome III questionnaire may be helpful even below the age of four.

To determine the most effective way to gather information on early GI symptoms in FD, this study compared three methods of assessing GI symptoms in infants and young children with *GLA* variants: pediatrician notes; the aforementioned FD-specific, parent-completed ROS questionnaire; and the parent-completed Rome III questionnaire. While a few studies have investigated the pediatric onset of symptoms in Fabry disease, this is the first to compare multiple means of assessing gastrointestinal symptoms in this population.

## Methods

### Data collection

Data utilized in this study was a subset of the data collected for a longitudinal, prospective, multicenter pilot study of clinical and biochemical findings of FD in young pediatric patients (the MOPPet study). See Laney et al., 2024 for detailed methods on recruitment, consent, and data collection beyond the scope of this paper. Briefly, participants 36 months or younger with *GLA* variants were recruited across the United States from 2014 to 2019. All participants were either diagnosed by family screening in a family with a known diagnosis of FD or through state-based newborn screening programs. Thus, the first states to implement newborn screening for FD are better represented in our study population. At the initiation of enrollment, Missouri was the only state in the United States conducting newborn screening for FD. Recruitment sites were the Emory University Lysosomal Storage Disease Center, Mercy Children’s Hospital, the University of Iowa, the University of Missouri, and Washington University in St. Louis. Genotypes included one variant of uncertain significance (VUS), p.Ala143Thr (A143T), classic and nonclassic variants for FD. The significance of the A143T variant has been debated. A spectrum of classifications have been proposed, including classic, nonclassic, benign, and pseudodeficiency variant [19, 20].

The specific data used in this study were collected from the Rome III and ROS questionnaires, both annually mailed to and completed by parents, and pediatrician notes gathered from participants’ local health care providers. The Rome III questionnaire is a validated and thorough questionnaire designed to assess functional gastrointestinal disorders available in the literature [21]. The gastrointestinal section of the ROS questionnaire asks about the presence and frequency and/or severity of abdominal pain, diarrhea, vomiting, and nausea. It also includes one question on maximum bowel movements per day and one question on stool consistency in the last week. The first version of this questionnaire did not ask about bloating and constipation, but these symptoms were added in a later version based on early parent feedback. The ROS questionnaire is not as detailed as the Rome III and is unpublished but available in our supplementary materials.

For each year, one pediatrician note was reviewed to record which of the examined GI symptoms were reported. Pediatrician notes were ideally a standard well-child visit, had to include a review of systems including GI, and could not be from a genetics or metabolic specialist. The note chosen was from the visit with the date closest to when the questionnaires were filled out. The rationale of using generalist rather than specialist notes was a) for consistency as not all participants were followed by a specialist and b) to increase the number of visits available to choose from. Ideal surveillance of GI symptoms would extend to generalist provider visits to increase the frequency of monitoring.

Gastrointestinal symptoms were documented by these three “methods of detection” over time. Each year of data was referred to as one “timepoint.” There could be multiple timepoints for a study participant if they had been enrolled for multiple years at the time of the study and returned multiple questionnaires. Each timepoint could include one of each questionnaire and one pediatrician note, or some combination of the three if one method of detection was missing. Since the goal of this study was a comparison, the analysis only included study participants who had at least one timepoint with multiple methods of detection available.

For analysis, where multiple questions addressed one symptom on the ROS or Rome III questionnaires, the questions were combined into a single variable that indicated whether a given symptom was detected by a given questionnaire. The constipation variable included questions about hard stools and the diarrhea variable included questions about soft stools. Symptom detection by the Rome III was always determined by reviewing multiple questions related to a symptom due to the detailed nature of the questionnaire.

### Statistical analysis

Survival curve analysis was based on the earliest ages at which GI symptoms were detected in each participant and graphed on a Kaplan-Myer Curve. Curves for each data collection method were compared using log rank test.

Kappa statistic was used to determine level of agreement between two methods as to whether a symptom was present at a particular timepoint for a particular individual. It was calculated for each symptom as well as for all GI symptoms combined. Data was separated to compare two data collection methods at a time to maximize how many timepoints could be used in each analysis. Kappa analysis was done for classic variant timepoints and A143T variants separately. Where parents did not mark a response on a questionnaire, it was counted the same as a response indicating an absent symptom. For timepoints where parents were given the earlier version of the ROS questionnaire which did not ask about constipation or bloating, these questionnaires were excluded from the appropriate analyses since parents did not have the opportunity to respond. To maximize the available data, no questionnaires were excluded from the “All GI symptoms” analyses. Because of the small n after breaking out data by method, variant, and age, Fisher’s exact test was used to compare symptom detection on the Rome III to detection on the ROS questionnaire within two age groups: 0–23 month age data vs 24+ month age data. Our sample size was too small to conduct meaningful analyses on smaller age subgroups or to further breakdown the subgroups by gender.

## Results

Of the 40 participants recruited to the MOPPet study, this analysis included 29 participants from 23 families. The other 11 participants were not included due to missing questionnaire data and limited availability of medical records. Data was considered “missing” if parents left unanswered fields on a questionnaire, did not return a questionnaire, or did not sign a medical release. Three participants were on enzyme replacement therapy at some point during their study participation; 26 were not. Demographic characteristics are summarized in Table 1. Age at participants’ first timepoint ranged from 3 to 49 months with a mean of 20.70 months (standard deviation 12.45) and a median of 21 months. The total number of timepoints analyzed was 114. The number of timepoints per participant ranged from 1 to 8 with a mean of 3.97 timepoints (standard deviation 2.20) and a median of 4 timepoints. The time between questionnaires and corresponding pediatrician note dates ranged from 0 to 169 days with a mean of 50.41 days (standard deviation 38.97) and a median of 42 days.

**Table 1:** Demographics of study participants

Total Participants		29
*GLA* Variant Type	Classic	13
	Nonclassic	5
	A143T	10
	VUS	1
Sex Assigned at Birth	Males	24
	Females	5
Race or ancestry	White	28
	White & Asian	1
Enzyme Replacement Therapy (at least one timepoint)	Yes	3
	No	26
Age at First Timepoint	0–5 months	4
	6–11 months	5
	12–17 months	1
	18–23 months	6
	2 years	8
	3 years	4
	4 years	1

Overall, GI symptoms were detected by at least one of the three methods in 53 of 60 timepoints for participants with classic variants (88%), 12 of 15 timepoints from participants with nonclassic variants (80%), 27 of 37 timepoints from participants with A143T variants (73%), and 2 of 2 timepoints from the participant with a VUS variant (100%).

Notably, question B16 from the Rome III questionnaires asks, ‘In the last year, how many times did your child have an episode of severe intense pain around the belly button that lasted 2 hours or longer and made your child stop everything that he or she was doing?’ This question identified nine timepoints in which a participant had experienced severe abdominal pain that hindered all activity and lasted at least two hours in the past year. Three of the nine timepoints were from the same participant in different years; the other six were each different participants. Eight of the nine timepoints were from participants with classic FD; one had an A143T variant. At each of these timepoints, abdominal pain was reported on the corresponding ROS questionnaire if available. In each case, abdominal pain was not recorded in the corresponding pediatrician’s note. Bloating was never reported in pediatrician notes for any participant.

**Fig. 1:**
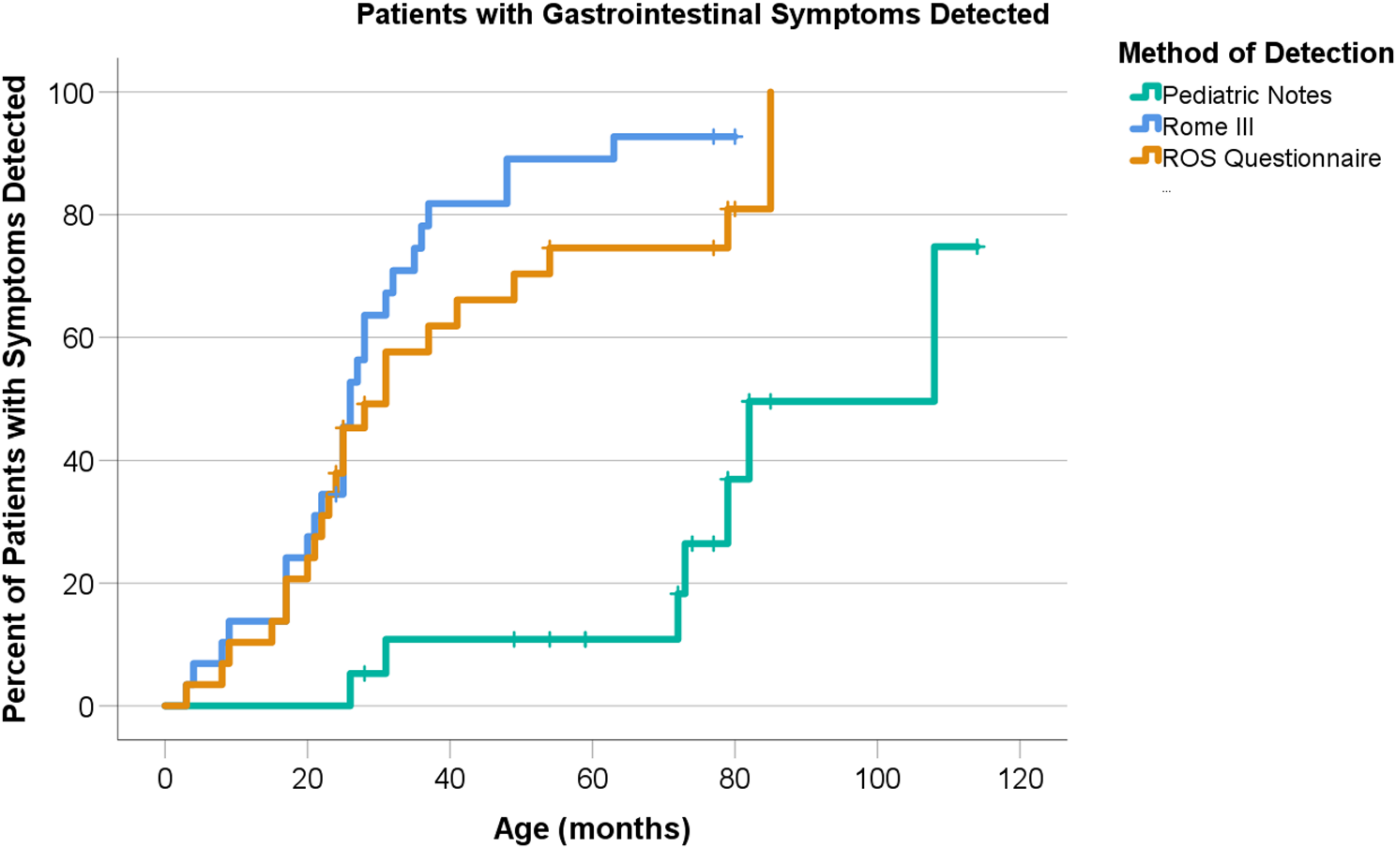
Questionnaires detected symptoms at an earlier age than pediatrician notes. Kaplan-Meier curve representing first gi symptom detection in each patient by three methods of detection. Crosses represent censored data: the last timepoint for a given participant where data from that method of detection was available

For the Kaplan-Meier curve in Fig. 1, the log-rank test showed a significant difference between pediatrician notes and Rome III (Chi-Square = 26.086, *p* < 0.001). The log-rank test also showed a significant difference between pediatrician notes and the ROS questionnaire (Chi-Square = 14.540, *p* < 0.001). However, there was no significant difference between Rome III and the ROS questionnaire (Chi-Square = 1.806, *p* = 0.179).

**Fig. 2:**
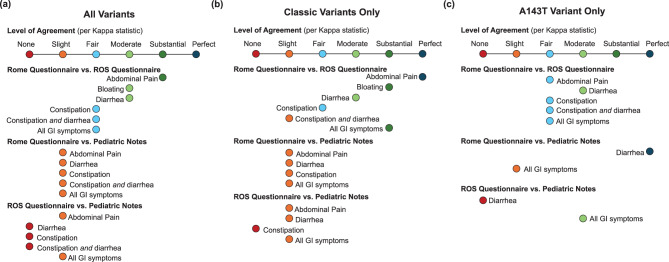
Graphical representation of level agreement between methods of data collection. Representation of agreement per kappa statistic on the presence of a given gi symptom for (**a**) all variants, (**b**) classic variants only, and (**c**) A143T variant only. Two methods of detection were compared at a time to maximize the number of timepoints that could be used. The highest groups available for comparison were for comparisons between the Rome III and ROS questionnaire. The lowest ns were for comparisons between ROS questionnaire and pediatrician notes. If a symptom was never detected by one method in a comparison, kappa statistic could not be calculated

Kappa statistic calculations are illustrated in Fig. 2 and the numerical values can be found in the Supplemental Materials. Overall, they showed stronger agreement between Rome III and the ROS questionnaire and weaker agreement between the pediatrician notes and either questionnaire. Most comparisons between the ROS questionnaire and the pediatrician notes had no agreement. Most comparisons between Rome III and pediatrician notes had slight agreement. Most comparisons between Rome III and the ROS questionnaire had fair to substantial agreement. Comparing Rome III to the ROS questionnaire, there was stronger agreement for abdominal pain and diarrhea and weaker agreement for constipation. There was limited data available on bloating. Agreement was somewhat weaker in the A143T variant group as compared to the classic variant group. In general, weaker agreement corresponded to the comparisons with smaller sample sizes, such as comparisons involving pediatrician notes (*n* = 13–68) and the A143T variant-specific analyses (*n* = 13–30). Comparisons for symptoms of constipation or bloating also had smaller sample sizes due to missing data.

Of the 113 ROS questionnaires, 16 (14.2%) did not ask about bloating and were excluded from the bloating Kappa and Fisher’s exact test analyses. Similarly, 72 (63.7%) of the ROS questionnaires did not ask about constipation so were excluded from the Kappa and Fisher’s exact test constipation and alternating constipation/diarrhea analyses.

Fisher’s exact test was used to compare symptom-by-symptom detection by Rome III to detection by the ROS questionnaire for two age groups: up to 23 months, and 24 months or older. Fisher’s exact test could not be calculated for the constipation symptom or to compare pediatrician notes to the questionnaires due to a small n. The remaining symptoms that were analyzed were: abdominal pain, diarrhea, all GI symptoms, and bloating. In the younger age group, there was no significant difference between the two questionnaires for these symptoms. However, there was a significant difference in the older age group (*p* < 0.001).

Because the prior analyses suggested that the added detail of the Rome III increased detection of GI symptoms in the older age group, a proposed questionnaire was designed for pediatric FD patients over the age of 2 years to collect more information than the ROS questionnaire without the extraneous detail of the Rome III. Manual analysis determined which of the Rome III questions allowed detection of additional symptoms in additional participants and which did not. For example, the two questions A2A and B1 together, but not separately, were sufficient to detect all instances of abdominal pain detected by Rome III. Question A2A asks about pain above the belly button, whereas B1 asks about pain around or below the belly button. Question B16 did not increase the number of instances in which abdominal pain was detected, but did add qualitative value because it asks about episodes of severe pain that lasted more than two hours. Therefore, variants of all three questions were included in the proposed questionnaire (Table 2, questions 1–3). Similar reasoning was used for each GI symptom. The final questions about vomiting, nausea, and early satiety were included, even though they were not part of the other analyses, because they are a significant part of the clinical picture despite being less prevalent [22].

**Table 2:** Proposed questionnaire. Eleven questions for the proposed questionnaire. All questions followed by “Yes” and “No” checkboxes, and further options such as “If yes, how often?: Daily, Weekly, Monthly, > monthly” or “Never, Once in a while, Sometimes, Always, I don’t know” are provided as appropriate. See Supplemental Materials for formatted, two-page questionnaire

Pediatric Gastrointestinal Symptom Questionnaire for Fabry Disease (24+ months)
1. In the last two months, did your child have any pain in the area above the belly button?
2. In the last two months, did your child have any pain in the area around or below the belly button?
3. In the last year, did your child have any episodes of severe intense pain around the belly button that lasted 2 hours or longer and made your child stop everything that they were doing?
4. In the last two months, has your child had any bloating?
5. In the last two months, how often did your child develop a clearly swollen belly during the day (you could see it was swollen)?
6. Has your child had diarrhea in the last two months?
7. In the last two months, how often were your child’s poops softer and more mushy or watery than usual?
8. Has your child been constipated in the last two months?
9. In the last two months, how often did your child have to strain (push hard) to make a poop come out?
10. Since your last visit, did your child have vomiting or nausea that was not caused by infection?
11. Since your last visit, has your child had a feeling of not being hungry after eating very little?

## Discussion

Optimizing care for patients with rare diseases is difficult. Over 7000 rare diseases exist, but most medical professionals will never see more than one case of a given rare disease in their careers. Specialist-created resources can assist non-specialists, such as primary care providers, in monitoring patients’ ever-changing health. This study identified gaps in symptom monitoring and created a preliminary resource for detecting pediatric GI symptoms in one rare condition: Fabry disease.

Early recognition and characterization of GI symptoms in FD is necessary to optimize individual monitoring and treatment plans. GI symptoms are a predominant feature of early FD. Both abdominal pain and diarrhea are common in FD and more prevalent in children than adults [4, 8, 22]. These symptoms can be severe but tend to improve with ERT or other treatments [15]. Similar symptoms experienced outside of a FD diagnosis receive treatment, even at a young age [23]. Detection of GI symptoms can shape both GI symptom management and treatment aimed at slowing overall progression of the disease. Since guidelines of when to begin ERT are based on an individual’s symptom onset and tailored to an individual’s symptom assessment [7, 8], monitoring for the earliest symptoms possible is crucial. Our cohort was heterogeneous and had small sample sizes. The scope of this paper was not to make claims about the prevalence of GI symptoms in different FD genotypes, although that has been explored using the MOPPet data in Laney et al., 2024. Rather, our aim was to compare and improve upon different methods of detecting GI symptoms. It is to be expected that pediatricians do not focus on monitoring for mild GI symptoms at each visit. However, involving pediatricians in the detection of new symptoms or progression of existing symptoms has the potential to characterize symptoms more clearly to convey to specialists. The need for primary care providers to be more involved in this way as part of the broader care team is something that has been proposed by other authors as well given the increased frequency that they see patients compared to specialists in many cases [13, 14].

Our study showed that not all GI symptoms present are detected, documented, or discussed at pediatrician visits. Both the ROS and Rome III questionnaires outperformed pediatrician notes as a way of recording GI symptoms, as evidenced by the log rank test for the Kaplan-Meier curve (Fig. 1). Agreement per kappa between the pediatrician notes and each questionnaire was none or slight for every symptom, whereas agreement between the two questionnaires was almost always fair or stronger (Fig. 2). In addition, bloating was never recorded on pediatrician notes but was reported on both questionnaires. While bloating might be considered a less severe symptom, it may be one of the earliest symptoms to present in FD and thus is an important symptom to address at wellness checks for these patients. Additionally, the deficiencies of pediatrician notes were not limited to mild symptoms. Severe abdominal pain is a symptom of FD that significantly affects quality of life and can be improved with treatment [22]. However, severe abdominal pain lasting more than two hours and hindering activity within the past year was never mentioned in pediatrician notes despite being reported nine times on Rome III. These types of symptoms may not be discussed in an appointment without providers asking more specific questions. Despite the limitation of questionnaires and pediatrician visits not occurring concurrently, noting that a patient has experienced severe abdominal pain between visits is highly relevant to their care. Severe pain should be addressed and managed by a physician. While outside the scope of this study, following up with these families to further assess their level of concern for these symptoms and how they were managed could provide meaningful context.

In the general population, a high level of detail regarding GI symptoms is not necessary during a pediatric well visit. It is well known that GI symptoms are nonspecific and highly prevalent in the general population [24]. This is likely one reason that fewer symptoms were recorded in pediatrician notes in this study. It is also possible that if the entire medical records were reviewed, additional GI symptoms would have been recorded in other notes. The intention of our method of using only one note per timepoint was to capture a snapshot of participants’ symptoms where the three methods could be reasonably compared. Ideally, the pediatrician note would have been from the same day as the questionnaires. Using notes from months earlier or later could capture different instances of GI symptoms as compared to what was documented on the questionnaires. Unfortunately, most participants did not have a pediatrician note available from the day or week of the questionnaires. But the difference in detection between the pediatrician notes and questionnaires was still pronounced.

Despite the prevalence of GI symptoms in the general population, in the context of pediatric patients with known FD there is more value in detecting these symptoms and thus in asking more detailed questions. These patients may consider initiating treatment based on multiple factors including their noted symptoms. For patients with *GLA* variants of uncertain significance identified on newborn screening, close monitoring for symptoms may also contribute to a diagnosis. In these situations, alternative methods to increase detection of symptoms, such as questionnaires, are warranted. Questionnaires filled out by parents are somewhat limited by recall bias, which might have been countered through the use of an ongoing symptom log. However, information gathered from parents in a pediatrician visit typically carries the same bias, without the advantages of asking more consistent or detailed questions.

Rome III detected more GI symptoms than the ROS questionnaire, but this was only statistically significant over 24 months of age (Fisher’s Exact Test, *p* < 0.001). Participants in the older group had a higher prevalence of symptoms. Additionally, older participants are more able to communicate discomfort, and some Rome III questions are difficult to apply to children in diapers [25]. All these factors could contribute to the age-related trend. The data suggests that the simpler ROS questionnaire is sufficient for children under 24 months. While Rome III detected the most GI symptoms, its length is prohibitive for practical, regular use and not all questions were necessary to elicit key symptoms. After 24 months, a FD-specific “key question” questionnaire is warranted.

Agreement between the ROS and Rome III questionnaires varied by symptom. There was weaker agreement between the two for constipation; more detailed questions may be warranted if the goal is to detect constipation and loose stools. However, over 60% of timepoints and participants had constipation or loose stools by the criteria we set for the Rome III answers, even within the nonclassic and A143T variant groups. The pathogenicity of the A143T variant is controversial, but not expected to cause symptoms in childhood with the majority of patients exhibiting a nonclassic phenotype [26, 27]. Additionally, constipation is highly prevalent in the general pediatric population [28, 29]. For these reasons, the higher detection rates of constipation or loose stools on Rome III as compared to the ROS questionnaire might not be valuable when the goal is to separate out affected and unaffected individuals. Rather, focusing on GI symptoms other than constipation could have more utility in that context. The trends observed regarding constipation were further limited since over half the copies of the ROS questionnaire did not ask about constipation, resulting in smaller sample sizes for constipation-related analyses.

Based on the above comparisons, we created a questionnaire for pediatric FD with the intention of maximizing symptom detection while remaining succinct. This questionnaire was not validated but is presented here as an example of a tool that could aid with detection of early GI symptoms and inform treatment plans earlier in pediatric patients with FD. Asking more detailed questions at primary care appointments would increase documentation and physician knowledge of patient GI symptoms at more frequent time points than the annual metabolic specialist appointments which are often annual. This would emulate models for other pediatric GI illnesses such as irritable bowel syndrome, where the use of Rome III criteria has helped to clarify uncertainty around a diagnosis [30]. Our data suggests using the ROS questionnaire before 24 months of age and a FD-specific questionnaire in pediatric patients starting at 24 months of age. The proposed example questionnaire can be found in Table 2. Inconsistencies in responses to the Rome III and ROS questionnaires emphasize the difficulties in interpreting data from multiple questionnaires and the necessity of a single, targeted FD symptom-specific questionnaire.

A pediatric FD-specific GI questionnaire would help fill a gap in monitoring very young children diagnosed with FD who do not routinely or frequently see metabolic specialists. It is particularly important as new states, such as Georgia and South Carolina, are considering adding FD to their state newborn screening. In these states, further guidelines are needed for monitoring going forward, as is collaboration between families and healthcare providers looking for early symptoms [31, 32]. Resources outlining specific and quantifiable ways to detect and report the earliest FD symptoms and when to follow-up with a gastrointestinal specialist are needed for these families. Future directions for research include validating the proposed FD GI symptom questionnaire, improving symptom elicitation guidelines in very young children with FD, and developing additional questionnaires for monitoring other early symptoms of FD such as neuropathic pain, heat intolerance, and cold intolerance.

There were several limitations to this study. In rare disease research, reaching statistically significant and generalizable sample sizes is often a challenge and was amplified here by the plan for prospective data. Recruiting participants under the age of 36 months was only possible at institutions that already followed families with a *GLA* variant or in states doing newborn screening for FD. At enrollment, Missouri was the only state in the country that included FD on newborn screening; this has now increased to 8 of 50 states. Even when recruited, medical records were not available for all patients or, if available, were not always complete. As per study design, questionnaires were filled out by parents and often had missing or conflicting data. However, this challenge would persist in real-world applications. Based on small numbers and the longitudinal nature of the study, some patients were overrepresented in the data because they had been in the study for longer, returned more questionnaires, or we had better access to their medical records. Additionally, the Rome III questionnaire was current in when data collection began and was used throughout the study for the sake of consistency. However, the Rome IV has several updates including distinguishing between toilet-trained and non-toilet-trained children regarding constipation; thus, the Rome IV questionnaire might be used in younger children more effectively. Finally, we recognized that in some countries, all patients with FD are monitored closely by a metabolic center at all ages and general pediatricians may play less of a role in assessing gastrointestinal symptoms in a pediatric cohort.

## Conclusion

To the best of our knowledge, this is the first study to compare multiple means of assessing gastrointestinal symptoms in a pediatric cohort of patients with Fabry disease. Although early GI symptoms have been described in Fabry disease before, in order to understand them better and monitor appropriately, the right tool is needed. Our comparison of existing tools emphasizes that not enough of these symptoms from the literature are being recorded, so a new strategy is needed. We conducted a prospective, longitudinal study including 29 young children with Fabry disease and were able to identify the areas where targeted questionnaires can increase our recognition of early symptoms as compared to a standard pediatrician visit. Additionally, we used this data as a foundation for building a newly proposed questionnaire. Early detection of symptoms in this population is critical as individual treatment plans are based on symptom onset and newborn screening for Fabry is available in several states and countries. We hope that these findings will inspire collaboration between metabolic specialists and primary care physicians leading to improved care for pediatric metabolic patients.

## Electronic supplementary material

Below is the link to the electronic supplementary material.

Supplementary Material 1

Supplementary Material 2

Supplementary Material 3

Supplementary Material 4

## Data Availability

The data that support the findings of this study are available from the corresponding author upon reasonable request.

## Abbreviations

A143T: The genetic variant *GLA* p.Ala143Thr
ERT: Enzyme replacement therapy
FD: Fabry disease
GI: Gastrointestinal
GL3: Globotriaosylceramide
ROS: Review of systems
VUS: Variant of uncertain significance
