# Supplementary material for: Targeted questionnaires improve detection of early gastrointestinal symptoms in young children with Fabry disease
Source: Orphanet J Rare Dis. 2026 Jan 20;21:21. doi: 10.1186/s13023-025-04168-3 (PMC12821958; doi:10.1186/s13023-025-04168-3)
Supplement: Supplementary file 1 — Supplementary Material 1 [file 13023_2025_4168_MOESM1_ESM.docx]

**Guide to Supplemental Data**

Documents:

- Guide to Supplemental Data.docx
  - Supplemental Table 1: Baseline Demographics of Enrolled Subjects (pages 1-2)
    - Includes specific variants and method of diagnosis. For method of diagnosis, NBS = newborn screening and FamHX = family history screening, i.e. there was a known diagnosis of Fabry disease in the family at the time of the participant’s birth.
  - Criteria for a symptom being “detected” on Rome III (page 2)
- Proposed Questionnaire (24+ months).pdf
  - Formatted version of proposed questionnaire
- ROS Questionnaire (under 24 months).pdf
  - Full ROS questionnaire that was used, reformatted
- Supplemental Table 2.xlsl
  - Kappa analyses with numerical values and sample sizes

| **Supplemental Table 1- Baseline Demographics of Enrolled Subjects** | | | | | | | |  |
| --- | --- | --- | --- | --- | --- | --- | --- | --- |
| ID | Gender | Race/Ethnicity | Variant | Classification | alpha gal levels nmol/hr/mg (Leukocyte) | Method of diagnosis | Baseline Age (months) | |
| **01-001** | **male** | White/Non-hispanic | c.1024C>T / p.R342* | Classic | 0.9 | FamHX | 11 | |
| **01-008** | **female** | White/Non-hispanic | c.777delA/ p.G261fs*8 | Classic | 20.3 | FamHX | 49 | |
| **01-009** | **male** | White/Non-hispanic | c.777delA/ p.G261fs*8 | Classic | 0.2 | FamHX | 21 | |
| **01-011** | **female** | White/Non-hispanic | c.982G>C / p.G328R | Classic | n/a | FamHX | 21 | |
| **01-012** | **male** | White/Non-hispanic | c.1042insG/p.A348Gfs*27 | Classic | n/a | FamHX | 7 | |
| **01-014** | **male** | White/Non-hispanic | c.999+2T>C/IVS6+*2T*>*C* | Classic | 0 | FamHX | 31 | |
| **01-021** | **female** | White/Non-hispanic | c.776C>G /p.P259R | Classic | n/a | FamHX | 8 | |
| **01-025** | **female** | White/Non-hispanic | c.777delA/ p.G261fs*8 | Classic | n/a | FamHX | 25 | |
| **01-029** | **male** | White/Non-hispanic | c.1024C>T /p.R342* | Classic | n/a | FamHX | 1 | |
| **01-033** | **male** | White/Non-hispanic | c.151C>T /p.R49C | Classic | n/a | FamHX | 21 | |
| **01-034** | **male** | White/Non-hispanic | c.777delA/ p.G261fs*8 | Classic | n/a | FamHX | 10 | |
| **01-035** | **female** | White/Non-hispanic | c.777delA/ p.G261fs*8 | Classic | n/a | FamHX | 3 | |
| **01-051** | **male** | White/Non-hispanic | c.679C>T /p.R227* | Classic | 1.1 | FamHX | 28 | |
| **01-023** | **male** | White/Non-hispanic | c.781G>A / p.G261S | NonClassic | n/a | FamHX | 17 | |
| **01-030** | **male** | White/Non-hispanic | c.870G>A /p.M290I | NonClassic | n/a | NBS | 16 | |
| **01-031** | **male** | White/Non-hispanic | c.870G>A / p.M290I | NonClassic | n/a | NBS | 36 | |
| **02-008** | **male** | White/Non-hispanic | c.369+5G>T/IVS2+5G>T | NonClassic | 3.6 | NBS | 28 | |
| **03-003** | **male** | White/Non-hispanic | c.335G>A /p.R112H | NonClassic | 0.7 | NBS | 25 | |
| **01-018** | **male** | White/Non-hispanic | c.427G > A / p.A143T | A143T | n/a | NBS | 21 | |
| **01-004** | **male** | White/Non-hispanic | c.427G > A /p.A143T | A143T | n/a | NBS | 5 | |
| **01-005** | **male** | White/Non-hispanic | c.427G > A /p.A143T | A143T | n/a | FamHX | 6 | |
| **01-019** | **male** | White/Non-hispanic | c.427G > A /p.A143T | A143T | n/a | NBS | 5 | |
| **01-027** | **male** | White/Non-hispanic | c.427G > A / p.A143T | A143T | n/a | NBS | 22 | |
| **02-006** | **male** | White/Non-hispanic | c.427G > A / p.A143T | A143T | 12.2 | NBS | 25 | |
| **02-010** | **male** | White/Non-hispanic | c.427G > A /p.A143T | A143T | 0.007 U/L | NBS | 26 | |
| **03-004** | **male** | White/Non-hispanic | c.427G > A /p.A143T | A143T | n/a | NBS | 2 | |
| **03-007** | **male** | White/Non-hispanic | c.427G > A /p.A143T | A143T | n/a | NBS | 4 | |
| **03-008** | **male** | White/Non-hispanic | c.427G > A /p.A143T | A143T | 12.8 | NBS | 3 | |
| **03-001** | **male** | White/Non-hispanic | c.593T>C / p.I198T | VUS | 8.5 | NBS | 26 | |

**Criteria for a symptom being “detected” on Rome III:**

Symptom detection by the Rome III was determined by reviewing the following cited responses to specific questions.

Bloating was “detected” if A2C was answered 1 or A11, B10, or D3 was answered 1-4.

Abdominal Pain was “detected” if A2A was 1, or B1 or B16 was 1-4.

Diarrhea was “detected” if A7 or B6 were 1-4 or C2 was 4 or 5.

Constipation was “detected” if A8, A10, B7, B9, C5, or C9 was 1-4 or C1, C8, or C10 was 1, or C2 was 1 or 2.
