# Supplementary material for: Targeted questionnaires improve detection of early gastrointestinal symptoms in young children with Fabry disease
Source: Orphanet J Rare Dis. 2026 Jan 20;21:21. doi: 10.1186/s13023-025-04168-3 (PMC12821958; doi:10.1186/s13023-025-04168-3)
Supplement: Supplementary file 3 — Supplementary Material 3 [file 13023_2025_4168_MOESM3_ESM.pdf]

ROS Questionnaire (GI Section) – under 24 months

**Abdominal Pain**	No	Yes		
		If yes:		
		-Frequency<br>Daily	Monthly	> Monthly
		- Intensity<br>Mild	Severe	Extreme
		- Severity past week<br>None	Moderate	Severe
**Bloating**	No	Yes		
		If yes:		
		Mild	Severe	
**Diarrhea**	No	Yes		
		If yes:		
		-Frequency<br>Daily	Monthly	> Monthly
**Constipation**	No	Yes		
		If yes:		
		-Frequency<br>Daily	Monthly	> Monthly
**Max # of bowel movements per day in past week:**	1-2	3-4	8-10	> 10
**Stool consistency on average during past week**	Very hard	Hard	Loose	Watery
**Vomiting**	No	Yes		
		If yes:		
		-Frequency<br>Daily	Monthly	> Monthly
**Nausea**	No	Yes		
		If yes:		
		-Frequency<br>Daily	Monthly	> Monthly
