# Supplementary material for: Targeted questionnaires improve detection of early gastrointestinal symptoms in young children with Fabry disease
Source: Orphanet J Rare Dis. 2026 Jan 20;21:21. doi: 10.1186/s13023-025-04168-3 (PMC12821958; doi:10.1186/s13023-025-04168-3)
Supplement: Supplementary file 4 — Supplementary Material 4 [file 13023_2025_4168_MOESM4_ESM.pdf]

Pediatric Gastrointestinal Symptom Questionnaire for Fabry Disease (24+ months)

1. In the last two months, did your child have any pain in the area above the belly button?	Yes ☐	No ☐
2. In the last two months, did your child have any pain in the area around or below the belly button?	Yes ☐	No ☐
3. In the last year, did your child have any episodes of severe intense pain around the belly button that lasted 2 hours or longer and made your child stop everything that they were doing?	Yes ☐	No ☐
4. In the last two months, has your child had any bloating?<br><br>*If yes, how often?*<br>☐ Once in awhile<br>☐ Sometimes<br>☐ Always<br>☐ I don't know	Yes ☐	No ☐
5. In the last two months, how often did your child develop a clearly swollen belly during the day (you could see it was swollen)?<br><br>☐ Never<br>☐ Once in awhile<br>☐ Sometimes<br>☐ Always<br>☐ I don't know		
6. Has your child had diarrhea in the last two months?<br><br>*If yes, how often?*<br>☐ Daily<br>☐ Weekly<br>☐ Monthly<br>☐ >Monthly	Yes ☐	No ☐
7. In the last two months, how often were your child's poops softer and more mushy or watery than usual?<br><br>☐ Never<br>☐ Once in awhile		

☐ Sometimes<br>☐ Always<br>☐ I don’t know		
8. Has your child been constipated in the last two months?<br><br>*If yes, how often?*<br>☐ Daily<br>☐ Weekly<br>☐ Monthly<br>☐ >Monthly	Yes<br>☐	No<br>☐
9. In the last two months, how often did your child have to strain (push hard) to make a poop come out?<br><br>☐ Never<br>☐ Once in awhile<br>☐ Sometimes<br>☐ Always<br>☐ I don’t know		
10. Since your last visit, did your child have vomiting or nausea that was not caused by infection?<br><br>*If yes, how often?*<br>☐ Daily<br>☐ Weekly<br>☐ Monthly<br>☐ >Monthly	Yes<br>☐	No<br>☐
11. Since your last visit, has your child had a feeling of not being hungry after eating very little?<br><br>*If yes, how often?*<br>☐ Daily<br>☐ Weekly<br>☐ Monthly<br>☐ >Monthly	Yes<br>☐	No<br>☐
